# Radiological Feasibility of the Endoscopic Transnasal Prelacrimal Recess Approach

**DOI:** 10.1055/s-0046-1820089

**Published:** 2026-09-15

**Authors:** Ahmed Ragab, Aymn Ali, Hossam Hussein, Abdeltwab Hassan, Shimaa Hassanein, Mohammad Noaman

**Affiliations:** 1 Department of Otorhinolaryngology, Menoufia University Faculty of Medicine, Shebeen El-Kom, Menoufia Governorate, Egypt; 2 Department of Radiology, Menoufia University Faculty of Medicine, Shebeen El-Kom, Menofia Governorate, Egypt

**Keywords:** maxillary sinus, functional endoscopic sinus surgery, computed tomography

## Abstract

**Background:**

Prelacrimal recess (PLR) distance, medial wall thickness, and the internal angle of the pyriform notch (APN) have been suggested by various studies as assessment metrics to determine the feasibility and intraoperative difficulty of the transnasal prelacrimal recess approach (TPRA), with ethnic variations.

**Objective:**

To assess the computed tomography (CT) scan feasibility of endoscopic TPRA in healthy and diseased paranasal sinuses

**Methods:**

Our sample consisted of 750 adults (1,500 sides) who had CT of the nose and paranasal sinuses (PNS) for various indications. The CT scans were analyzed to assess variations in the distance, thickness, and angle of the prelacrimal recess on both sides concerning different demographic parameters (age and gender). All parameters were grouped into low, median, or high radiological feasibility of TPRA.

**Results:**

There was no difference in feasibility parameters between 546 healthy PNS (72.8%) and 204 chronic rhinosinusitis cases (27.2%). Only 2.7% of the maxillary sinuses studied (1,500 sides) had high radiological feasibility, 53.1% had medium radiological feasibility, and 44.2% had low radiological feasibility for TPRA. Males and individuals younger than 30 years of age had a significantly greater distance and thinner walls, making the sinus more surgically accessible ( *p* < 0.05).

**Conclusion:**

The three parameters studied can be collectively applied to the preoperative assessment of patients subjected to TPRA with no effect of inflammation on accessibility parameters. Difficult accessibility can be anticipated in 42% of the population, so surgeons must have caution when practicing TPRA.

## Introduction

Multiple changes to endoscopic medial maxillectomy have been suggested; among them is making the prelacrimal recess (PLR) the entry point into the maxillary sinus , thus offering the advantage of preserving the inferior turbinate and nasolacrimal duct (NLD). The endoscopic transnasal prelacrimal recess approach (TPRA) provides the surgeon with better access to the anterior maxillary sinus and other structures, such as the pterygopalatine, infratemporal and middle cranial fossa, as well as the lateral recess of the sphenoid sinus. 1 2 3

The TPRA was found to be difficult in some patients; however, this difficulty could be predicted preoperatively to appropriately plan the operation. 4 5 6 Simmen et al. 7 were the first to measure the average distance between the pyriform aperture (PA) and the anterior part of the NLD. Longer distance allows easier accessibility. 8 The thickness of the medial bony wall of the PLR can also predict the surgical difficulty of TPRA. It is believed that the thinner the wall, the more accessible the maxillary sinus. 1 Another radiological parameter that can be estimated is the internal angle of the pyriform notch (APN), which can correlate to intraoperative difficulty, maneuverability of instrumentation, and the need to widen the surgical access. 9

There are differences in one or more of the three parameters among the populations studied. Most studies were conducted on Asian and western countries' populations. The TPRA was found to be feasible (type II or III) in > 90% of a representative sample from Eastern Asia, while the feasibility was < 70% in a sample from western countries. 7 10

Surgical accessibility parameters have not been studied collectively. Furthermore, accessibility difficulties are not known in healthy and chronic rhinosinusitis (CRS) patients from Mediterranean ethnic countries. Therefore, we aimed to study the radiological feasibility of TPRA in healthy and CRS patients in the Egyptian population using the three parameters, namely distance between PA and NLD, thickness of the medial bony wall of the PLR, and APN.

## Methods

The present study is a prospective, simple, randomized cross-sectional study of the PNS of healthy patients who had computer tomography (CT) scans of the nose and PNS for various indications and of CRS patients who were diagnosed according to the European Position Paper on Rhinosinusitis and Nasal Polyps (EPOS) 2020. 11 Patients were divided into 2 groups: group 1, without pathological findings; and group 2, with CRS.

The study was reviewed and approved by the Ethics Committee of the university hospital with the code (10/2023 ENT 31). All patients gave an informed consent after explanation.

All 750 patients underwent PNS CT scan examination with a SOMATOM scanner (Siemens Healthineers). Computed tomography scans were analyzed to evaluate variations in the distance, thickness and angle of the prelacrimal recess bilaterally in relation to different demographic parameters (age and gender). The measurements were read by a senior radiologist and an ear, nose, and throat (ENT) consultant with an intervariability measurement of 0.08 using a dedicated PNS protocol.

The distance between the front wall of the maxillary sinus and the nasolacrimal duct (the prelacrimal recess distance) was measured, and, based on this distance, the prelacrimal recess was classified into 3 types: type I (< 3 mm), type II (3–7 mm), and type III (> 7 mm). 7

At the same plane, we measured the APN between the anterior and medial maxillary walls. 10 The angle was also classified into type I (< 45°), type II (45–60°), or type III (> 60°). 12

The thickness of the medial maxillary wall was measured above the level of NLD, and was also classified into 3 types: type I (> 5 mm), type II (2–5 mm), and type III (< 2 mm).

The CT scans were analyzed to assess all three variations in the distance, thickness, and angle of the PLR on both sides using the following classification of different types of PLR.

**Fig. 1:**
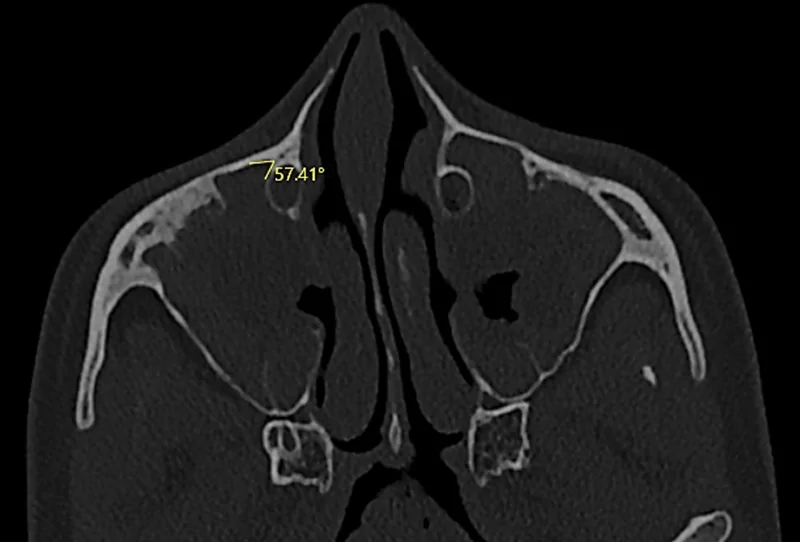
Computed tomography scan showing low grade feasibility of the transnasal prelacrimal recess approach (TPRA) in right chronic rhinosinusitis type Ia, with the yellow line representing the angle of the TPRA.

The feasibility of TPRA was determined as follows:

I. Low-grade feasibility ( Figs. 1 2 3 )
  - Type Ia: (type-I distance + type-I thickness + any angle)
  - Type Ib (type-I distance + type-II or -III thickness + any angle)
II. Medium-grade feasibility ( Figs. 4 5 )
  - Type IIa (type-II distance + type-I thickness + any angle)
  - Type IIb (type-II distance + type-II or -III thickness + any angle)
III. High-grade feasibility ( Fig. 6 )
  - Type IIIa (type-III distance + type-I thickness + any angle)
  - Type IIIb (type-III distance + type-II or -III thickness + any angle)

**Fig. 2:**
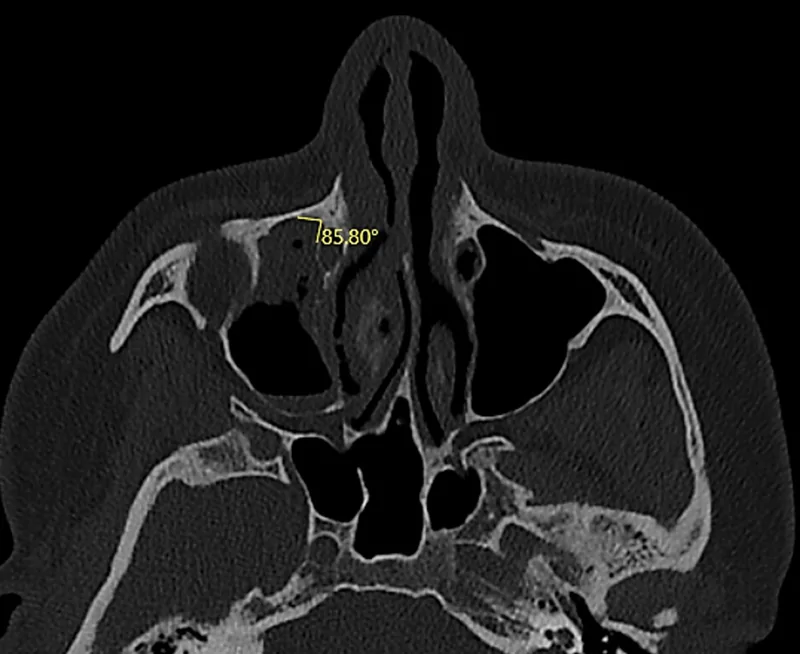
Computed tomography scan showing low grade feasibility of the TPRA in right CRS type Ib, with the yellow line representing the angle of the TPRA.

**Fig. 3:**
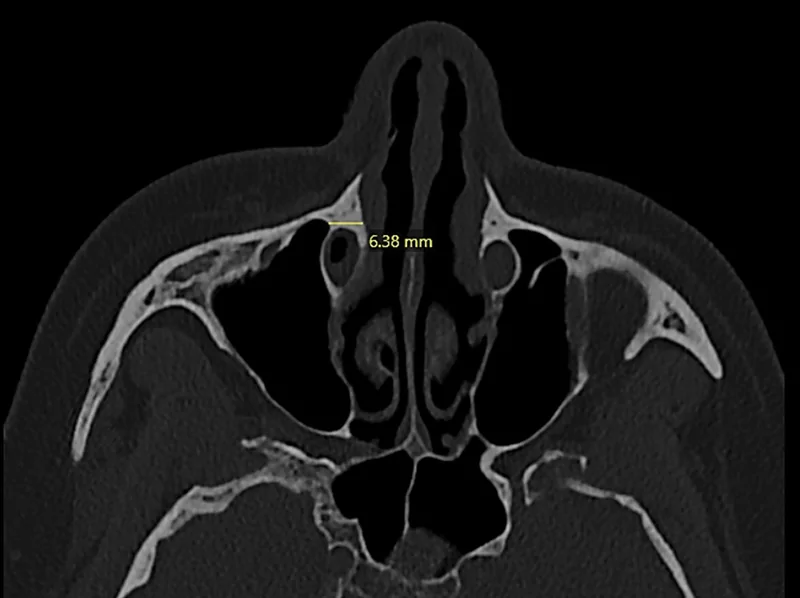
Computed tomography scan showing low grade feasibility of the TPRA in right healthy sinus type Ia, with the yellow line representing the thickness of the TPRA.

**Fig. 4:**
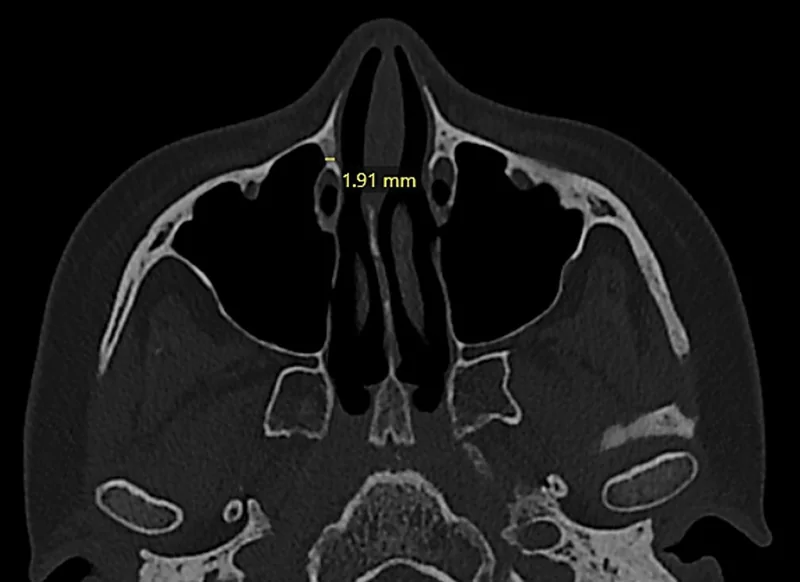
Computed tomography scan showing medium grade feasibility of the TPRA in right healthy sinus type IIb, with the yellow line representing the thickness of the TPRA.

**Fig. 5:**
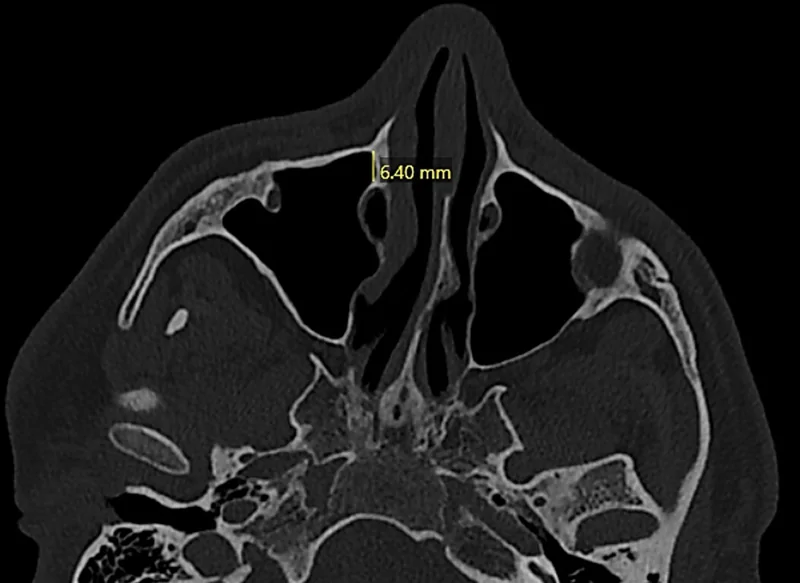
Computed tomography scan showing medium grade feasibility of the TPRA in right healthy sinus type IIb, with the yellow line representing the distance of the TPRA.

**Fig. 6:**
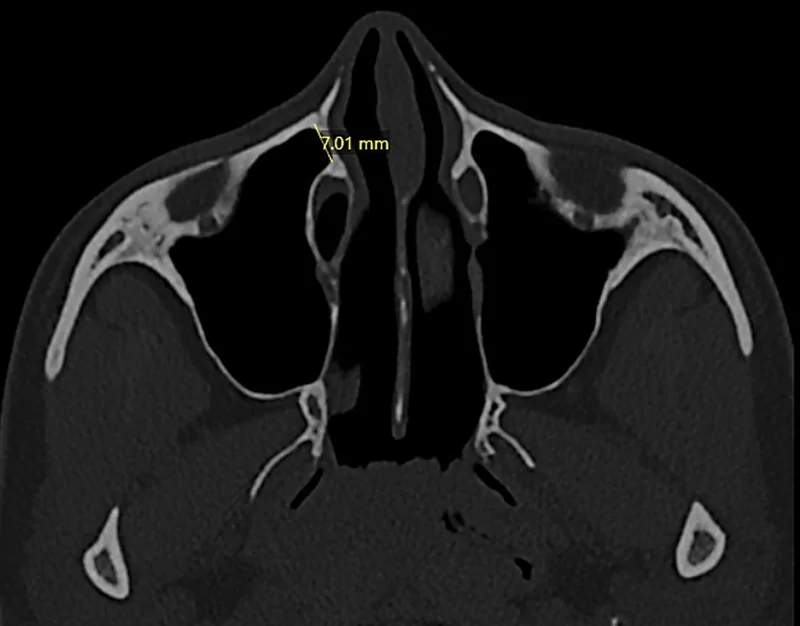
Computed tomography scan showing high grade feasibility of the TPRA in right healthy sinus type IIIb, with the yellow line representing the distance of the TPRA.

The data were analyzed in relation to each group and in relation to demographic data of age and sex.

### Statistical Analysis of the Data

Data were analyzed using an IBM personal computer with IBM SPSS Statistics for Windows (IBM Corp.), version 20.0, and Epi Info 2000 (public domain) software. Descriptive data were presented as mean ± standard deviation (SD). The Mann-Whitney test was used for comparison between two groups with non-normally distributed quantitative variables. The Spearman correlation coefficient test (r-test) was used to study the correlation between non-parametric quantitative variables. A *p* -value of (≤ 0.05) was considered statistically significant.

## Results

In the current study, CT scans of 750 adult patients (1,500 sides) were analyzed to evaluate the variations in the distance, thickness, and angle of the prelacrimal recess bilaterally in relation to different demographic parameters (age and sex). Group І involved 546 healthy PNS (72.8%) and group ІІ involved 204 sinuses with CRS, representing 27.2% of the study group ( Table 1 ).

**Table 1:** Descriptive data of the participants recruited

Item	Frequency (n = 750)	Percentage (%)
**Gender**		
Male	392	52.3
Female	358	47.7
**Age (years)**		
< 30	242	32.3
≥ 30	508	67.7
Mean ± SD	36.8 ± 13.8	
Range	18–85	
Median (IQR)	35 (25–45)	
**Maxillary sinus disease state**		
CT with pathological findings	204	27.2
CT without pathological findings	546	72.8

**Abbreviations:** CT, computed tomography; IQR, interquartile range; SD, standard deviation.

### The PLR Distance

The mean PLR distance for the total 750 patients (1,500 sides) was 3.38 ± 1.67 mm, with values ranging from 0 to 8.6 mm on both sides. The mean PLR distance, in both groups were 3.35 ± 1.69 mm and 3.41 ± 1.66 mm on the right and left sides, respectively.

The mean PLR distances in the healthy group were 3.36 ± 1.69 mm and 3.40 ± 1.68 mm on the right and left sides, respectively. The mean PLR distances in the CRS group were 3.33 ± 1.69 mm and 3.44 ± 1.60 mm on the right and left sides, respectively.

There were no statistically significant differences in the PLR distance between both sides and between the healthy and CRS groups ( *p* > 0.05) ( Tables 2 3 ).

**Table 2:** Radiological feasibility of the TPRA in relation to diagnosis in the participants recruited

Items	CRS patients (n = 204; 27.2%)		Healthy PNS (n = 546; 72.8%)		Test of significance; *p* -value
	n	%	n	%	
**Right prelacrimal recess distance type**					
**Type I**	88	43.1	245	44.9	χ ^2^ = 0.360; *p* = 0.835 (> 0.05)
**Type II**	111	54.4	285	52.2	
**Type III**	5	2.5	16	2.9	
**Left prelacrimal recess distance type**					
**Type I**	84	41.2	246	45.1	χ ^2^ = 0.926; *p* = 0.629 (> 0.05)
**Type II**	114	55.9	284	52	
**Type III**	6	2.9	16	2.9	
**Right prelacrimal recess thickness type**					
**Type I**	40	19.6	95	17.4	χ ^2^ = 1.55; *p* = 0.460 (> 0.05)
**Type II**	110	53.9	322	59	
**Type III**	54	26.5	129	23.6	
**Left prelacrimal recess thickness type**					
**Type I**	30	14.7	71	13	χ ^2^ = 0.470; *p* = 0.791 (> 0.05)
**Type II**	109	53.4	304	55.7	
**Type III**	65	31.9	171	31.3	
**Right prelacrimal recess angle type**					
**Type I**	3	1.5	1	0.2	χ ^2^ = 4.64; *p* = 0.098 (> 0.05)
**Type II**	4	2	11	2	
**Type III**	197	96.6	534	97.8	
**Left prelacrimal recess angle type**					
**Type I**	1	0.5	3	0.6	X ^2^ = 0.012; *p* = 0.994 (> 0.05)
**Type II**	4	2	11	2	
**Type III**	199	97.5	532	97.4	

**Abbreviations:** χ ^2^ , Chi-aquared test; CRS, chronic rhinosinusitis; PNS, paranasal sinuses; TPRA, transnasal prelacrimal recess approach.

**Note:** The *p* -values refer to the comparison between the two stidy groups.

**Table 3:** Radiological feasibility of the TPRA in relation to diagnosis in the participants recruited

Items	CRS patients (n = 204)		Healthy PNS (n = 546)		Test of significance; *p* -value
	n	%	n	%	
**Right prelacrimal recess distance**					
**Mean ± SD**	3.33 ± 1.69		3.36 ± 1.69		Mann-Whitney U = 0.058; *p* = 0.954 (> 0.05)
**Min.-max.**	0–8.5		0–8.5		
**Median (IQR)**	3.2 (2.2–4.5)		3.1 (2.2–4.4)		
**Left prelacrimal recess distance**					
**Mean ± SD**	3.44 ± 1.60		3.40 ± 1.68		Mann-Whitney U = 0.381 *p* = 0.703 (> 0.05)
**Min.-max.**	0–8.6		0–8.5		
**Median (IQR)**	3.3 (2.2–4.4)		3.15 (2.3–4.4)		
**Right prelacrimal recess thickness**					
**Mean ± SD**	3.39 ± 1.99		3.39 ± 1.85		Mann-Whitney U = 0.463; *p* = 0.643 (> 0.05)
**Min.-max.**	0.4–10.5		0.4–8.6		
**Median (IQR)**	2.9 (1.88–4.6)		3.1 (2–4.5)		
**Left prelacrimal recess thickness**					
**Mean ± SD**	2.99 ± 1.678		3.02 ± 1.676		Mann-Whitney U= 0.075; *p* = 0.941 (> 0.05)
**Min.-max.**	0.4–6.8		0.4–7.6		
**Median (IQR)**	2.8 (1.6–4.28)		2.7 (1.8–4)		
**Right prelacrimal recess angle**					
**Mean ± SD**	100.3 ± 20.03		104.1 ± 18.1		Mann-Whitney U= 2.10 *p* = 0.036* (≤ 0.05)
**Min.-max.**	42–140.5		44–151		
**Median (IQR)**	104 (85.5–115.2)		105 (92–116.5)		
**Left prelacrimal recess angle**					
**Mean ± SD**	100.3 ± 18.8		103.2 ± 19.6		Mann-Whitney U = 1.88; *p* = 0.061 (> 0.05)
**Min.-max.**	41.5–141		35–151		
**Median (IQR)**	102.3 (91–117)		100.7 (88.5–113)		

**Abbreviations:** CRS, chronic rhinosinusitis; IQR, interquartile range; Max., maximum; Min., minimum; PNS, paranasal sinuses; SD, standard deviation; TPRA, transnasal prelacrimal recess approach.

**Notes:** The *p* -values refer to the comparison between the two stidy groups; *statistically significant ( *p* ≤ 0.05).

A statistically significant difference in the PLR distance was found between male and female as well as between both age groups intervals on both sides ( *p* < 0.05).

According to the present study, 2.9% had a PLR distance > 7 mm (type III) and 52.9% with distance of 3 to 7 mm (type II), making the PLR surgically accessible, while 44.2% had a PLR distance of < 3 mm (type I), making the PLR approach in these cases technically difficult.

### The PLR Thickness

The mean PLR thickness in the total 750 patients (1,500 sides) was 3.2 ± 1.8 mm. While the mean PLR thickness in both groups on the right side was 3.39 ± 1.89 mm, and the mean on the left side was 3.01 ± 1.68 mm.

The mean PLR thicknesses in the healthy group were 3.39 ± 1.85 mm and 3.02 ± 1.676 mm on right and left sides, respectively. The mean PLR thicknesses in the CRS group were 3.39 ± 1.99 mm and 2.99 ± 1.678 mm on right and left sides, respectively.

A statistically significant difference in the PLR thickness was found between both sides ( *p* < 0.05). There is no statistically significant difference in the PLR thickness between healthy and CRS sinus ( *p* > 0.05).

While there was a statistically significant difference in the PLR thickness between male and female and between both age groups on both sides ( *p* < 0.05).

According to the present study, 27.9% of studied maxillary sinuses (1,500 sides) had PLR thickness < 2 mm (type 3) and 56.3% had a PLR thickness 2 to 5 mm (type II), making PLR surgically accessible, while 15.7% had PLR thickness of > 5 mm (type I), making TPRA in these cases technically difficult.

### The PLR Angle between the Anterior and Medial Maxillary Walls

The mean PLR angle in the 750 patients (1,500 sides) was 102.8 ± 19.06°. While the mean in both groups on the right side was 103.6 ± 18.7°, and the mean on the left side was 102.4 ± 19.4°.

The mean PLR angles in the healthy group were 104.1 ± 18.1° and 103.2 ± 19.6° on the right and left sides, respectively. The mean PLR angles in the CRS group were 100.3 ± 20.03° and 100.3 ± 18.8° on the right and left sides, respectively.

There was no statistically significant difference in the PLR angle between both sides or between male and female patients on both sides ( *p* > 0.05).

There was statistically significant difference in the PLR angle between both age groups on both sides ( *p* < 0.05).

According to the present study, 97.5% and 2% of the studied maxillary sinuses (1,500 sides) had a PLR angle > 60° (type III) and 45 to 60° (type II), respectively, while 0.5% had a PLR angle < 45° (type I).

According to the present study, 2.9% of the total maxillary sinuses studied (1,500 sides) had a high radiological feasibility (type III) and 52.9% had a medium radiological feasibility (type II), while 44.2% had a low radiological feasibility (type I) of TPRA ( Table 4 ).

**Table 4:** Distribution of the maxillary sinuses studied according to the feasibility of the TPRA

Type	Frequency (n = 1,500)
Low feasibility (Ia)	197 (13.1%)
Low feasibility (Ib)	466 (31.1%)
Medium feasibility (IIa)	38 (2.5%)
Medium feasibility (IIb)	756 (50.4%)
High feasibility (IIIa)	0 (0%)
High feasibility (IIIb)	43 (2.9%)

**Abbreviation** : TPRA, transnasal prelacrimal recess approach.

Pre-lacrimal recess distance and angle were negatively correlated with thickness. On the other hand, they were positively correlated with each other ( *p* < 0.001) ( *n* = 1,500).

## Discussion

**Fig. 7:**
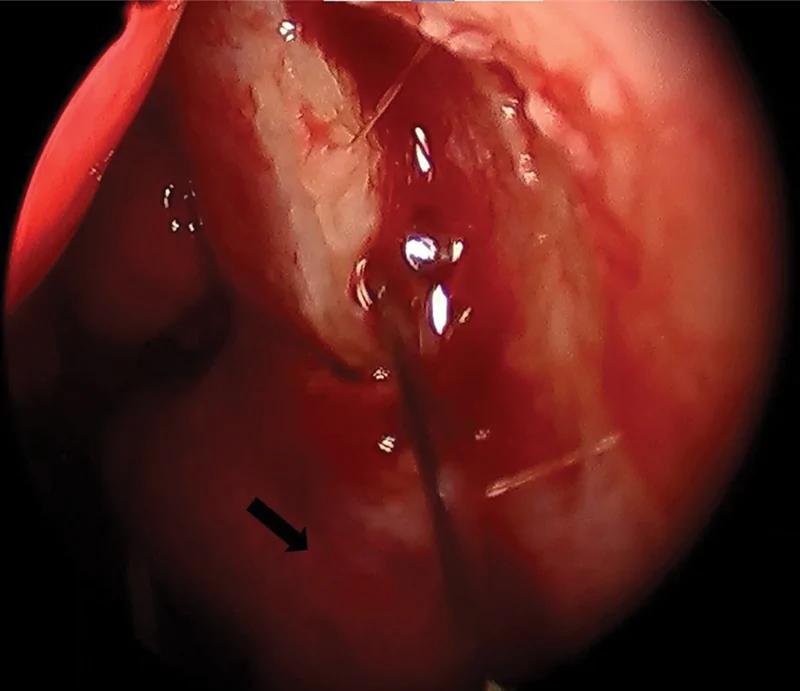
Intraoperative endoscopic view of the site of incision via the prelacrimal fossa.

Transnasal prelacrimal recess approach ( Fig. 7 8 ) to the maxillary sinus permits visualization and instrumentation of most inaccessible areas with minimum long-term complications. 13 14 The larger the distance between the PA and the anterior part NLD, the more accessible the sinus. 8 So, distance is crucial for planning the TPRA.

**Fig. 8:**
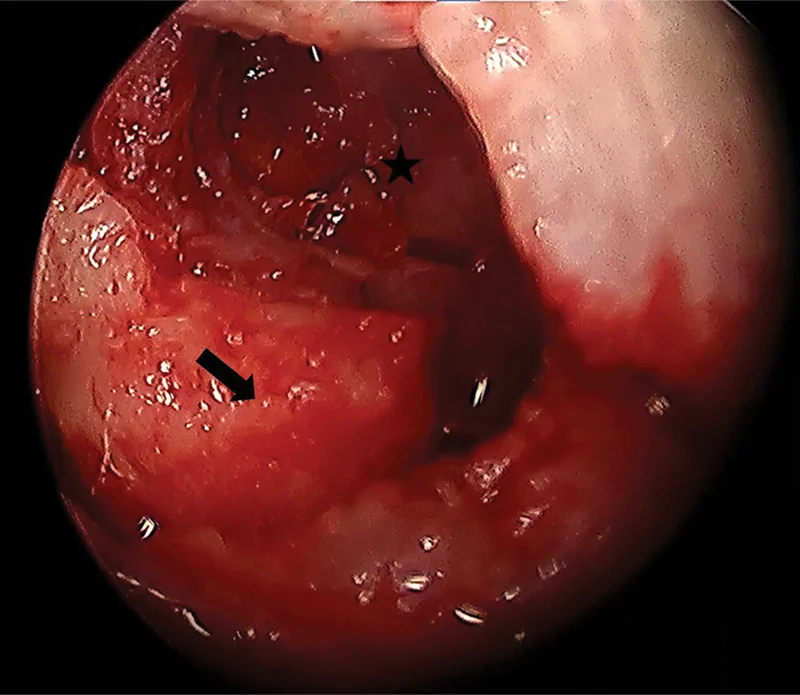
Intraoperative endoscopic view of the TPRA showing the landmarks for orientation of the approach, the black arrow denotes the inferior turbinate, and the black star indicates the maxillary sinus.

The distance was found to be different between studies, dependent on race, age, and sex of studied population. Simmen et al. 7 were the first to measure the distance in 100 Caucasians, and the mean distance from the anterior wall of the maxillary sinus to the NLD was 4.24 ± 2.40 mm. A similar distance was found in other studies in which the study population was mainly western in origin. Sieskiewicz et al. 15 found that the mean distance was 4.00 mm in the CT of 125 patients from Poland. Andrianaki et al. 16 (100 patient from Austria) found the mean distance to be 3.7 ± 2.5 mm.

On the other hand, Lock et al. 10 found the mean distance to be of 6.64 ± 2.36 mm in the CT of 100 Chinese patients. Chen et al. 1 (255 patients from China) found that the mean distance was 4.62 ± 1.74 mm.

In the current study, the mean PLR distance in the 750 Egyptian patients was 3.38 ± 1.67 mm, which is in line with the findings from western studies.

Different studies had significant differences in PLR distance between male and female. In agreement with the findings of the present study, Sieskiewicz et al., 15 Wang et al., 17 Kashlan et al., 8 and Andrianakis et al. 16 found that the mean PLR distance was significantly larger in males than in females, making TPRA more feasible in males. The difference between both genders could be explained by the larger anteroposterior dimension of the maxillary sinus in males when compared with females.

In contrast, Chen et al. 1 found the PLR distance to be similar between genders but the mean thickness of the medial bony wall of the PLR was significantly larger in males than in females. However, a significant negative correlation was found between thickness and distance of the PLR which agrees with the current results.

Simmen et al. 7 found that types I, II, and III cases made up 31.5%, 56%, and 12.5%, respectively which was similar to the results of other western studies. Lupu et al. 12 (Romania) have found that types I, II, and III cases made up 35%, 53.3%, and 11.66%, respectively. Andrianakis et al. 16 (Austria) found that types I, II, and III cases made up 38.5%, 49.5%, and 12%, respectively.

In contrast, the Asian population had more feasibility grades, Son et al. 18 has found that types I, II, and III cases made up 22.7%, 54.7%, and 22.5%, respectively. Koo et al. 19 (Korea) found that types I, II, and III cases made up 23.2%, 55.0%, and 21.8%, respectively.

In a study by Kim et al., 20 the feasibility of TPRA was 92.2% in Koreans, which was similar to that found in Chinese (93.0%) 10 but different from that of Europeans (68.5%). 7 So, TPRA could be more feasible in Northeast Asians.

In the current study, types-I, -II, and -III cases made up 44.2%, 52.9%, and 2.9% of the study population, respectively. Less feasibility is observed in the Egyptian population than in the European and Asian.

Another factor that was found to affect the feasibility of TPRA, and that also differs by gender, is prelacrimal recess thickness.

According to Chen et al., 1 the PLR's medial bony wall has a wide range of thicknesses, from 0.3 to 8.1 mm. This indicates that the PLR's medial bony wall may be very thick, requiring extensive bone removal to do TPRA, or it may be very thin, allowing for easy performance of the procedure with minimal bone removal. The mean thickness found by Chen et al. was 2.84 ± 1.41 mm, which similar to the one of the current study, which was 3.2 ± 1.8 mm (0.4–10.3 mm).

The last factor that was found to affect the feasibility of TPRA is APN. Arosio et al. 9 have found that the mean APN was 54° (28–73°). Lupu et al. 12 (Romania) have found the angle's mean value to be ∼ 80.8 ° (75.5–85.8 °). In the current study, the mean was 102.8 ± 19.06 °. The difference can be explained by the race of the samples studied.

A statistically significant difference in the PLR distance and thickness was found in the present study between both age groups ( *p* < 0.05), with the younger age group having more PLR distance, with thinner walls and more accessible sinuses.

Chen et al. 21 found that the prevalence of PLR declined with the increase in age with age-related decrease in the maxillary sinus volume. It was found that maxillary sinuses continue to develop until the 2nd and 3rd decades of life, and, after complete development, their volume decreases, which could explain why the maxillary sinus is wider in the younger age group.

In agreement with the findings of the present study, Simmen et al. 7 found no significant difference in the PLR distance between the left and right sides. This result was confirmed by Chen et al., 21 who discovered no significant difference in the medial bone wall thickness between the various groups. Wang et al. 17 did note that around 14.56% of individuals had inconsistent PLR on the left and right sides. The thickness of the PLR has also been shown to vary in certain patients between the left and right sides; however, this was not fully examined in that study. As a result, while contemplating bilateral endoscopic TPRA, comprehensive assessment of the anatomical structure distinctive in each side should be required. 1

## Conclusion

All three parameters studied can be collectively applied to aid preoperative assessment of the patients subjected to TPRA. High radiological feasibility of TPRA was found to be the least common in the Egyptian population, with no difference between healthy and CRS patients. Males and younger age groups had more PLR distance, thinner walls, and more accessible sinuses than females and older age group.
